# A genome-wide survey of the secondary metabolite biosynthesis genes in the wheat pathogen *Parastagonospora nodorum*

**DOI:** 10.1080/21501203.2014.928386

**Published:** 2014-06-24

**Authors:** Yit-Heng Chooi, Mariano Jordi Muria-Gonzalez, Peter S. Solomon

**Affiliations:** ^a^ Plant Sciences Division, Research School of Biology, The Australian National University, Canberra, 0200, Australia

**Keywords:** *Parastagonospora nodorum*, secondary metabolites, plant pathogen, polyketide synthase, nonribosomal peptide synthetase, terpene synthase

## Abstract

The model pathogen *Parastagonospora nodorum* is a necrotroph and the causal agent of the wheat disease Septoria nodorum blotch (SNB). The sequenced *P. nodorum* genome has revealed that the fungus harbours a large number of secondary metabolite genes. Secondary metabolites are known to play important roles in the virulence of plant pathogens, but limited knowledge is available about the SM repertoire of this wheat pathogen. Here, we review the secondary metabolites that have been isolated from *P. nodorum* and related species of the same genus and provide an in-depth genome-wide overview of the secondary metabolite gene clusters encoded in the *P. nodorum* genome. The secondary metabolite gene survey reveals that *P. nodorum* is capable of producing a diverse range of small molecules and exciting prospects exist for discovery of novel virulence factors and bioactive molecules.

## Introduction

Filamentous fungi are prolific producers of bioactive small molecules, known as secondary metabolites (SMs). Fungi are an important source for drug discovery, examples of which include important drugs like the antibiotic penicillin and the cholesterol-lowering statins; yet many SMs are mycotoxins that are harmful to humans, such as aflatoxins, trichothecenes and fumonisins. In plant pathogenic fungi, the SMs often play an important role in plant infection and virulence. Fungi are the major causal agents of diseases in crop plants. SMs that lead to the damage or killing of plants are also known as phytotoxins, and their modes of action are diverse (Möbius et al. 2009). These phytotoxins include plant-damaging photosensitizers (e.g. cercosporin) (Daub et al. 2005), inhibitors of plant cellular functions (e.g. ATPase inhibitor tentoxin) (Meiss et al. 2008), membrane disruptants (e.g. beticollin 0) (Goudet et al. 2000) or triggers of plant apoptosis (Möbius et al. 2009). Some of these phytotoxins are host specific while others have activities against a broader range of plant hosts. Some well-known SM host-specific toxins (HSTs) include HC-toxin (Walton 2006), victorin (Lorang et al. 2012) and T-toxins (Turgeon & Baker 2007). These HSTs are known to target plant hosts by interacting with a specific susceptibility gene in an inverse manner of the classical gene-for-gene system, similar to the proteinaceous effectors in necrotrophic pathogens (Oliver & Solomon 2010; Stergiopoulos et al. 2013).

Other SMs that are not directly involved in damaging the plants could play important role in protection against environmental stress (e.g. melanin) (Eisenman & Casadevall 2012), mineral uptake (e.g. siderophores) (Johnson 2008), interfering host hormone signalling (e.g. giberrellins) (Johnson 2008) and suppressing plant defence (e.g. supprescins and brefeldin) (Tietjen & Matern 1984; Wada et al. 1995). As individual pathogenic fungi and their hosts are not isolated in the environment, the pathogens will be constantly interacting with other organisms, including other competing microbes, endophytes and fungivores. Therefore, it is not surprising that many SMs from plant pathogenic fungi also possess antibacterial, antifungal and cytotoxic activities.

Although there are increasing numbers of SMs being isolated and characterized from plant pathogenic fungi, our understanding of SM production and their functional roles in these pathogens is still largely incomplete. This is especially true for many wheat pathogens, which are currently the focus of the research in our laboratory. Wheat (*Triticum aestivum*) is among the most important staple food crops. Like most crop plants, fungi are the biggest threat among the causal agents of diseases in wheat, causing significant yield loss annually. Among the most important wheat fungal diseases are the yellow (tan) spot caused by *Pyrenophora tritici-repentis*, Septoria nodorum blotch caused by *Parastagonospora nodorum*, Septoria tritici blotch caused by *Zymoseptoria tritici*, stripe rust caused by *Puccinia striiformis* f. sp. *tritici*, crown rot caused by *Fusarium pseudograminearum* and the head blight caused by *Fusarium graminearum* (Murray & Brennan 2009).

Recent advances in genome sequencing technology have led to an unprecedented surge of genomic information that was previously unavailable. These whole genome sequencing efforts have revealed that fungal genomes encode for immense biosynthetic potential that far surpasses the chemical diversity that we have previously perceived. This holds true as well for the major wheat pathogens mentioned above. The availability of genome sequences for all these wheat pathogens allows a preview to their genetic potential for SM biosynthesis and it shows that all of these wheat pathogens, except the rust (basidiomycete) fungus *Puccinia striiformis* f.sp. *tritici*, are rich in SM biosynthesis genes. This review will focus on the model pathogen *Parastagonospora nodorum*, a necrotroph that cause Septoria nodorum blotch (SNB) in wheat. Here, we review the SMs that are known to be produced by this fungus and provide an in-depth genomic examination of the SM biosynthesis genes in this wheat pathogen, which we hope to serve as the basis for future investigation into the secondary metabolome of *P. nodorum*.

## *Parastagonospora nodorum* pathobiology and secondary metabolites

**Figure 1.**
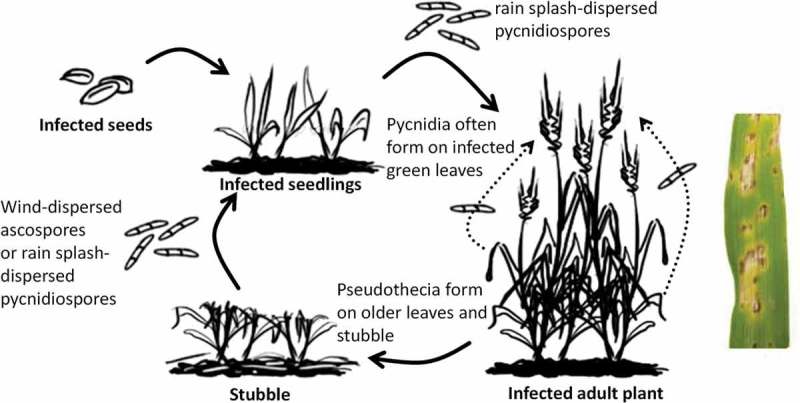
*Parastagonospora nodorum* causing Septoria nodorum blotch (SNB) on wheat. Left: the *P. nodorum* lifecycle. Right: the disease symptom of SNB on wheat leaf.

*Parastagonospora nodorum* falls within the order Pleosporales in the class Dothideomycetes (Oliver et al. 2012). More commonly known as *Stagonospora nodorum* Berk. (teleomorph *Phaeosphaeria nodorum*), this fungus has been recently re-named as *Parastagonospora nodorum* (Berk.) Quaedvlieg, Verkley & Crous in a molecular taxonomy study due to the clustering of the type of the fungal genus *Stagonospora* (*Stagonospora paludosa*) with Massarinaceae and not Phaeosphaeriaceae (Quaedvlieg et al. 2013). Furthermore, it was shown in the same study that *P. nodorum* does not cluster with or near the type of the genus *Phaeosphaeria* (*Phaeosphaeria oryzae*). SNB (Figure 1) leads to > $100 million yield loss per annum in Australia (Murray & Brennan 2009) and is one of the major plant pathogens in North America and worldwide. It is genetically tractable and has well-annotated genome sequence (Hane et al. 2007; Oliver et al. 2012). The availability of simple *in vitro* (detached-leaf) and whole plant (spray) virulence assays offer an excellent model for studying the functional roles of SMs in plant pathogens. We will discuss briefly the pathogen biology, lifecycle and modes of nutrition of *P. nodorum,* and how SMs may play a role in their different stages of growth.

*Parastagonospora nodorum* is a necrotrophic pathogen, thus its lifecycle can be divided into a parasitic and saprophytic stage (Figure 1). During the parasitic phase, it can infect all above-ground plant parts with the asexual pycnidiospores and sexual ascospores dispersed to upper plant parts, including wheat heads, primarily via rain splashes (Griffiths & Ao 1976; Brennan et al. 1985). However, the sexual ascospores produced by the perithecia are capable of long-distance wind dispersal (Sanderson & Hampton 1978; Bathgate & Loughman 2001). The ascospore-producing perithecia are present during both the parasitic and saprophytic phase. During the saprophytic phase, the pathogen overwinters on wheat straw and stubble until the parasitic phase starts again (Shaner 1981). Both infected seeds and ascospores are considered the major inocula of this fungal wheat disease (Solomon et al. 2006).

*Parastagonospora nodorum* produces proteinaceous necrotrophic effectors as major pathogenicity factors, where each interacts with a specific wheat susceptibility gene, not unlike the gene-for-gene relationship observed in plant disease resistance to biotrophs (Oliver et al. 2012). Three such effectors, SnTox1, SnToxA and SnTox3, which induce necrosis in specific genotype of wheat, have been identified from *P. nodorum* (Oliver et al. 2012). Recent studies suggest that there may be more necrotrophic effectors in *P. nodorum* that remain to be identified (Francki et al. 2011; Crook et al. 2012; Friesen et al. 2012; Tan et al. 2013).

**Figure 2.**
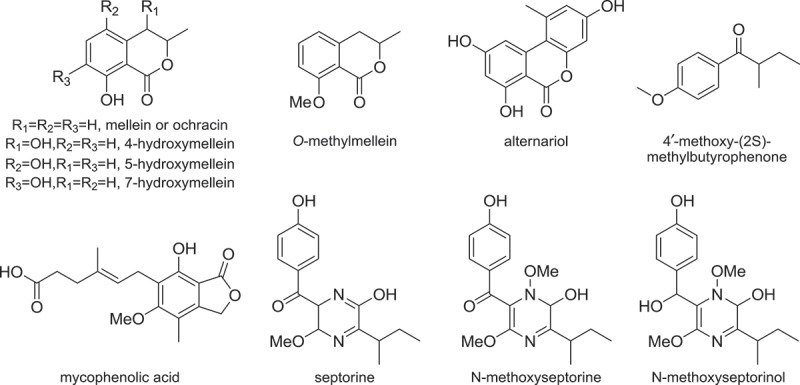
Secondary metabolites isolated from *Parastagonospora nodorum.*

Despite the importance of *P. nodorum* among wheat diseases, the SM repertoire of this fungus is poorly understood. The SM molecules that have been identified so far include septorines (Devys et al. 1978, 1982, 1992), melleins and mycophenolic acids (Devys et al. 1980, 1994) (Figure 2). Even though Devys et al. have continually worked on isolating SMs from *P. nodorum* for over a decade, apparently only analogues of these three above groups of compounds from *P. nodorum* can be identified via traditional compound isolation approaches. It is possible that *P. nodorum* only produces these compounds above as major metabolites under the laboratory culture conditions tested whilst other metabolites are either not produced or only present in minor quantities. This is not surprising given that many fungal SM pathways are known to be silent and only expressed in response to specific environmental stimuli (Brakhage & Schroeckh 2011).

More recently, alternariol has been identified in a metabolomics study of a *P. nodorum* mutant lacking a short-chain dehydrogenase (*Sch1)* (Tan et al. 2009). The study found that the concentration of alternariol in the culture of an *sch1* mutant strain is 200-fold greater than the wild-type *P. nodorum*, although the relationship between *sch1* and alternariol biosynthesis is still unknown. Another new compound, (+)-4ʹ-methoxy-(2S)-methylbutyrophenone (Figure 2), was isolated from *P. nodorum* recently by supplementing epigenetic modifiers to the culture medium (Yang et al. 2013). These two most recent *P. nodorum* SM studies have highlighted that both genetic and epigenetic approaches can be used to induce the expression of otherwise silent SM pathways in laboratory culture conditions (Brakhage & Schroeckh 2011).

Among the known *P. nodorum* SMs, mellein and septorines have been shown to exhibit phytotoxic activities (Venkatasubbaiah & Chilton 1990; Parisi et al. 1993). To date, no SM-type effector has been identified in *P. nodorum*. The SMs in *P. nodorum* are most likely to have auxiliary roles in virulence against wheat, but the presence of such host-specific effectors cannot be excluded (Syme et al. 2013). It can also be speculated that SMs are likely to play various other roles, such as in fungal development (e.g. sporulation) and inter-species competition (e.g. during saprophytic stage), at the different stages of *P. nodorum* lifecycle.

Since limited information is available for SMs from *P. nodorum,* we have surveyed the literature for compounds produced by fungi of the same genus (*Phaeosphaeria* or *Stagonospora*) to obtain some insights into the diversity of SM compounds that might be produced by *P. nodorum* (see Supplemental Table 1). The literature survey revealed that species in the related genera are capable of producing diverse structures with the SM compounds dominated by polyketides followed by a small number of terpenoid compounds and alkaloids. Some of these compounds will be related to the *P. nodorum* SM biosynthetic genes to be discussed in the following section.

**Table 1.** SM backbone enzymes found in *Parastagonospora nodorum* genome.

SM backbone enzymes	
Polyketide synthase	23
Non-reducing PKS	7
Partially reducing PKS	1
Highly reducing PKS	14
Hybrid PKS-NRPS	1
Type III PKS	1
Nonribosomal peptide synthetase	14
Multimodular NRPS	6
Dimodular NRPS	1
Monomodular NRPS-like	7
Sesquiterpene synthases (class I)	3
Diterpene synthase (class II)	1
DMATS-type Prenyltransferase	2
UbiA-like Prenyltransferase	3

## Genomic outlook for the SM biosynthetic potential of *Parastagonospora nodorum*

The identification of the genes involved in fungal SM pathways has been partly facilitated by the likelihood that genes belonging to a single pathway will cluster together on chromosomes (Hoffmeister & Keller 2007). The recent progress in our understanding of the molecular genetics of fungal secondary metabolism has been further propelled by the advances in genomic technologies with an increasing number of SMs now being linked to their biosynthetic genes or gene clusters. Consequently, we now have the ability to identify the genes within fungal genomes that encode for SM biosynthetic enzymes and provide clues to the structural class of the compounds that may be produced based on the type of backbone biosynthetic enzymes. For example, the structurally diverse polyketides are produced by the large multifunctional iterative type I polyketide synthases (PKSs), which utilize acetate/malonate units as building blocks. The carbon backbones of aromatic compounds, such as aflatoxins and griseofulvin, are synthesized by non-reducing PKSs (NR-PKSs), while the aliphatic compounds (acyclic and cyclic), such as fumonisins and lovastatin, are produced by highly reducing PKSs (HR-PKSs) (Cox 2007; Chooi & Tang 2012). Besides iterative Type I PKSs, some fungi also harbour Type III PKSs, which are more commonly found in plants (e.g. chalcone synthase) (Seshime et al. 2005). In contrast, the non-ribosomal peptide compounds, such as the cyclicpeptides HC-toxin and echinocandins, are produced by large multimodular enzymes called non-ribosomal peptide synthases (NRPSs), which utilize proteinogenic and non-proteinogenic amino acids as building blocks (Finking & Marahiel 2004). Terpenoids/isoprenoids, on the other hand, are synthesized from multimers of five-carbon isoprene units assembled by isopropanoid synthases (ISs) and prenyltransferases (PTs) folded by various terpene synthases (TSs) into diverse structures (Christianson 2008), e.g. trichothecenes, botrydial. Fungal SMs can also be produced from mixed pathways; for example, tenuazonic acid and cytochalasin are generated from polyketide-nonribosomal peptide pathways, whereas sirodesmin is produced from nonribosomal peptide-isoprenoid pathway and fumagillin originates from polyketide-isoprenoid pathways. Typically, the backbone SM genes are clustered with multiple genes encode for tailoring enzymes such as oxygenases, methyltransferases, acyltransferases, etc., that further diversify the chemical structures.

The genome sequencing of *P. nodorum* SN15 was completed in 2007 and was the first fungal genome in the large Dothideomycete class to be sequenced (Hane et al. 2007). More recently, the resequencing and comparative genomics of *P. nodorum* have further improved the assembly of this genome and revealed additional SM genes after sequence correction (Syme et al. 2013). The 37 Mb genome of *P. nodorum* encodes 23 PKSs, 14 NRPSs, 4 TSs and 5 PTases (Table 1). More than one SM backbone biosynthetic genes are found in some of the *P. nodorum* SM gene clusters, forming hybrid gene clusters, thus the total number of SM gene clusters is lower than the number of SM backbone biosynthetic genes. SMURF SM gene cluster prediction software detected a total of 29 SM gene clusters in *P. nodorum* (Khaldi et al. 2010). However, we can identify a total of 38 SM gene clusters in *P. nodorum* by including the TSs, type III PKSs, and UbiA-like PTases not considered in the SMURF software and some PKS and NRPS genes left out in the analysis (Supplemental Table 2). Nevertheless, only five groups of SM molecules have so far been identified in *P. nodorum* (Figure 2), and they therefore only represent about 13% of the SM biosynthesis potential of *P. nodorum*. Furthermore, none of these identified SMs have been linked to the genes in *P. nodorum.*

**Table 2.** Domain architecture of PKSs encoded in *Parastagonospora nodorum* genome.

PKS locus	Domain architecture	Closest characterized BLAST hit/polyketide product
**NR-PKSs**		
SNOG_06682	SAT-KS-AT-PT-ACP-CM-TE/CLC	*Penicillium brevicompactum* MpaC (38%)/mycophenolic acid
SNOG_07020	SAT-KS-AT-PT-ACP-ACP-CM-TE	*Monascus purpurus* CtnPKS (29%)/citrinin
SNOG_08274	SAT-KS-AT-PT-ACP-ACP-TE/CLC	*Nectria haematococca* PKSN (52%)/unknown red pigment
SNOG_08614	SAT-KS-AT-PT-ACP-TE/CLC	*Cercospora nicotianae* CTB1 (56%)/cercosporin
SNOG_09932	SAT-KS-AT-PT-ACP-TE/CLC	*Aspergillus nidulans* wA (42%)/naphthopyrone YWA1
SNOG_11981	SAT-KS-AT-PT-ACP-ACP-TE/CLC	*Alternaria alternata* ALM1 (80%)/melanin
SNOG_15829	SAT-KS-AT-PT-ACP	*Penicillium aethiopicum* GsfA (63%)/griseofulvin
**HR-PKSs**		
SNOG_02561	KS-AT-DH-CM-ER-KR-ACP	*Phoma* sp. SQTKS (35%)/squalestatin tetraketide
SNOG_04868	KS-AT-DH-CM-ER-KR-ACP	*Leptosphaeria maculans* PKS2 (90%)/phomenoic acid
SNOG_05791	KS-AT-DH-CM-ER-KR-ACP	*Alternaria solani* PKSN (82%)/alternapyrone
SNOG_06676	KS-AT-DH-ER-KR-ACP	*Alternaria brassicicola* DEP5 (66%)/depudecin
SNOG_07866	KS-AT-DH-CM-KR-ACP-R	*C. heterostrophus* PKS16 (74%)/uncharacterized
SNOG_09490	KS-AT-DH-ER-KR-ACP	*Botryiotinia fuckeliana* BcBOA9 (38%)/botcinic acid
SNOG_09623	KS-AT-DH-CM-KR-ACP	*Fusarium verticillioides* Fum1p (40%)/fumonisin
SNOG_11066	KS-AT-DH-ER-KR-ACP	*L. maculans* LEMA_P006610 (54%)/uncharacterized
SNOG_11076	KS-AT-DH-CM-ER-KR-ACP	*Fusarium verticillioides* Fum1p (38%)/fumonisin
SNOG_11272	KS-AT-DH-ER-KR-ACP	*Botryitis cinerea* BcBOA9 (50%)/botcinic acid
SNOG_12897	KS-AT-DH-ER-KR-ACP	*Chaetomium chiversii* CcRADS1 (45%)/radicicol starter unit
SNOG_13032	KS-AT-DH-CM-ER-KR-ACP	*Fusarium verticillioides* Fum1p (38%)/fumonisin
SNOG_14927	KS-AT-DH-KR-ACP	*Fusarium verticillioides* Fum1p (41%)/fumonisin
SNOG_15965	KS-AT-DH-ER-KR-ACP	*Fusarium verticillioides* Fum1p (35%)/fumonisin
**PKS-NRPS**		
SNOG_00308	KS-AT-DH-CM-ER°-KR-ACP-C-A-T-R	*Aspergillus clavatus* CcsA (46%)/cytochalasin E and K
**PR-PKSs**		
SNOG_00477		*Aspergillus terreus* 6MSAS (54%)/6-methylsalicylic acid
**Type III PKSs**		
SNOG_09622	KS-AT-TH-KR-ACP	*Neurospora crassa* ORAS (36%)/2ʹ-oxoalkylresorcylate

Notes: PKS domain abbreviation – SAT, starter unit; ACP transacylase; KS, β-ketoacyl synthase; MAT, malonyl-CoA:ACP transacylase; PT, product template; ACP, acyl-carrier protein; TE/CLC, thioesterase/Claisen cyclase; CM, *C*-methyltransferase; DH, dehydratase; ER, enoyl reductase; KR, ketoreductase; TH, thiohydrolase. See Chooi and Tang (2012) review for detail description of the PKS functional domains. Closest BLAST hit is included when there is no characterized PKS in the top 100 hits.

### Polyketide synthases (PKSs)

All the common types of fungal type I PKSs (NR-, HR- and PR-PKSs) as well as the less common type III PKSs can be found in *P. nodorum* (Table 1). The number of PKS genes (total 24) in *P. nodorum* is comparable to those found in the genome of *Aspergillus* spp., which are often regarded as highly prolific SM producers (Sanchez et al. 2012). Some of these *P. nodorum* PKSs showed significant homology to characterized PKS genes, inferring that they may produce compounds of similar structures. Other PKSs though have the domain architecture and/or accessory enzymes encoded in their gene clusters that match the biosynthetic requirements required to make certain compounds isolated previously from *P. nodorum* and related species. The domain architectures of the PKSs encoded by these genes and their corresponding homologues (closest characterized homologue and/or closest BLASTP homologue) are shown in Table 2. We will discuss selected PKS (NR-PKS, HR-PKS, PR-PKS, type III PKS) genes/gene clusters with the aim to provide an overview of the chemical structural diversity encoded in the genome of *P. nodorum.* In each section, the genes are listed in the order from those having the highest similarity (at the protein level) to another known gene, followed by those with more tentative matches.

**Figure 3.**
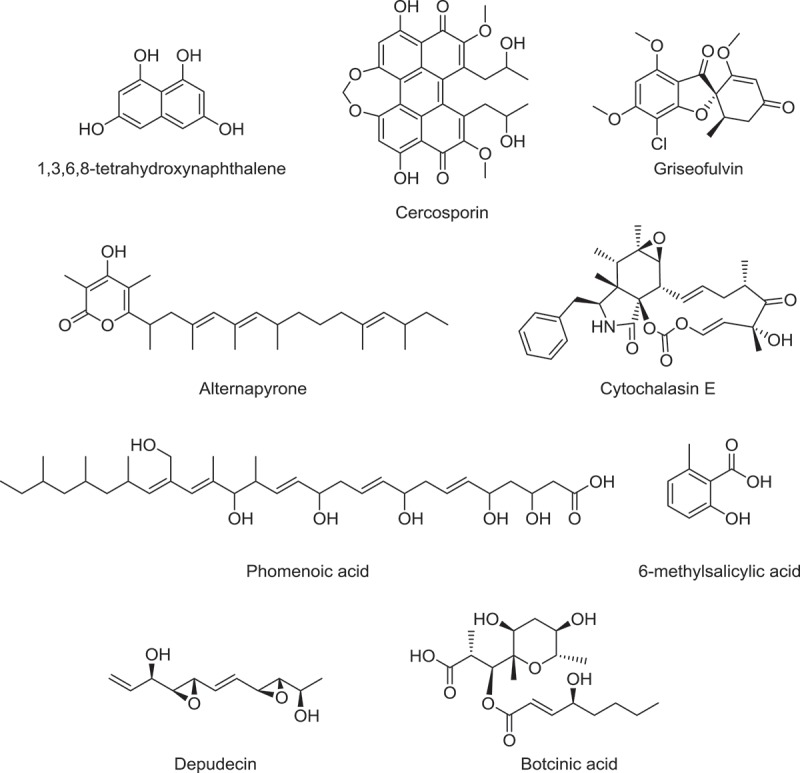
Polyketide compounds synthesized by putative homologues of *Parastagonospora nodorum* PKSs.

Among the NR-PKSs, SNOG_11981 shares 80% protein identity with the *Alternaria alternata* ALM1, which is responsible for biosynthesis of DHN (1,8-dihydroxynaphthalene) melanin precursor 1,3,6,8-tetrahydroxynaphthalene (Figure 3) (Kimura & Tsuge 1993), suggesting that it may play a similar role in *P. nodorum*. The biosynthesis of DHN melanin may proceed either via the pentaketide route followed by deacetylation (by NR-PKSs such as *Colletotrichum lagenarium* PKS1) or via the heptaketide route followed by chain-shortening (by NR-PKSs such as *Aspergillus nidulans* WA and *Aspergillus fumigatus* Alb1) to form the key precursor 1,3,6,8-tetrahydroxynaphthalene (Tsai et al. 2001; Fujii et al. 2004; Wheeler et al. 2008; Vagstad et al. 2012). The similarity of SNOG_11981 and *A. alternata* ALM1 to *C. lagenarium* PKS1 (46% and 47% identity, respectively) and *A. nidulans* WA (44%% and 46% identity, respectively) are comparable, thus it remains to be determined if *P. nodorum* and *A. alternata* biosynthesize melanin via the pentaketide or heptaketide pathway. Interestingly, *P. nodorum* has also been shown to synthesize melanin via the L-DOPA (l-3,4-dihydroxyphenylalanine) pathway (Solomon et al. 2004). Other NR-PKSs that may involve in pigment biosynthesis are SNOG_08274 and SNOG_09932, which are homologues of the *Nectria haematococcoa* PKSN involved in red perithecia pigment biosynthesis (Graziani et al. 2004) and *A. nidulans* WA heptaketide napthopyrone synthase (Fujii et al. 2001), respectively (Table 2). It is yet to be determined if SNOG_08274 or SNOG_09932 is responsible for the pink pigmentation of *P. nodorum* conidia during their extrusion from the pycnidia.

Another *P. nodorum* NR-PKS with significant similarity to characterized PKSs is SNOG_15829, which is 63% identical to griseofulvin PKS GsfA from *Penicillium aethiopicum –* the closest characterized homologue (Chooi et al. 2010). Like GsfA, SNOG_15829 lacks the C-terminal thioesterase (TE) releasing domain often required for product release either by Claisen cyclization or hydrolysis (Crawford & Townsend 2010; Chooi & Tang 2012). Most of these ‘TE-less’ NR-PKSs require a standalone metallo-β-lactamase-like TE for releasing the mature polyketide products (Awakawa et al. 2009; Li et al. 2011). Nonetheless, GsfA alone is capable of catalysing both aldol cyclization and Claisen cyclization of a heptaketide intermediate and release norlichexanthone as the immediate product (Cacho et al. 2013). The heptaketide chain length of griseofulvin (Figure 3) corresponds to the polyketide backbone of the mycotoxin alternariol (Thomas 1961), suggesting that SNOG_15829 is a promising candidate for alternariol production in *P. nodorum*.

*Parastagonospora nodorum* also contains a NR-PKS (SNOG_08614) that shares 56% identity to cercosporin synthase CTB1 from *Cercospora nicotianae* (Choquer et al. 2005). Homologues of O-methyltransferase CTB2 in the cercosporin pathway can also be found in the vicinity of SNOG_08614 locus (Chen et al. 2007) (Supplemental Table 2, cluster 19). Cercosporin (Figure 3) is a host non-selective photoactivated phytotoxin that has been demonstrated to contribute to the virulence of the plant pathogenic *Cercospora* species (Daub 1982; Daub et al. 2005). Similar perylenequinone compounds (compound **27-40** in Supplemental Table 1) have been isolated from an endolichenic *Phaeosphaeria* sp. (Li et al. 2012), but not in *P. nodorum*. Whether *P. nodorum* produces such perylenequinone compounds and their involvement in pathogenesis of wheat are questions that warrant further investigation.

SNOG_06682 and SNOG_07020 belong to a subclass of NR-PKSs that contain a C-methyltransferase domain. Both NR-PKSs also contain a TE domain at the C-terminal, instead of the thioreductase domain commonly found in azaphilone-type PKS gene clusters (Zabala et al. 2012). NR-PKSs with similar domain architecture to SNOG_06682 and SNOG_07020 have been implicated in biosynthesis of dimethylorsellinic acid (DMBA)-type meroterpenoids (Itoh et al. 2012; Lo et al. 2012) as well as mycophenolic acid (Figure 3) (Hansen et al. 2011, 2012; Regueira et al. 2011). Hansen et al. (2012) noted that *P. nodorum* SNOG_06682 gene cluster shared multiple homologues with the mycophenolic acid *mpa* cluster (*mpaC, mpaD* and *mpaE*) in *Penicillium brevicompactum*, suggesting that both clusters shared a common ancestry (Supplemental Table 2, cluster 13). Interestingly, mycophenolic acid has been reported to be isolated from *P. nodorum*. However, SNOG_06682 shares only 38% identity with the PKS MpaC in the *mpa* cluster and the corresponding gene cluster does not contain a UbiA-like membrane-bound long-chain prenyltransferase MpaA that was assumed to transfer a farnesyl chain to the phthalide intermediate. Instead, the SNOG_06682 gene cluster encodes a prenyltransferase belonging to the DMATS (dimethylallyl tryptophan synthase)-type short-chain prenyltransferase family, which are soluble α/β-fold proteins (Li 2009). A HR-PKS gene (SNOG_6676) is also found in vicinity of SNOG_06682 NR-PKS gene. It is unknown if the sequenced *P. nodorum* SN15 strain produces mycophenolic acid using a slightly different enzyme repertoire or if it produces a structural analogue of mycophenolic acid.

Among the *P. nodorum* HR-PKSs, SNOG_05791 shared 82% identity with the alternapyrone synthase PKSN from *Alternaria solani* (Fujii et al. 2005). However, alternapyrone (Figure 3), the direct product of PKSN, is unlikely to be the final product of the gene cluster as three cytochrome P450 genes and one oxidase gene are found in the vicinity of *pksN* (Fujii et al. 2005). Likewise, a flavin-dependent oxidase gene and a cytochrome P450 gene can be found in the vicinity of SNOG_05791; the two clusters may produce similar products. Since *A. solani* is also a plant pathogen (causes tomato and potato early blight disease), it would be interesting to examine whether the products of these two clusters are implicated in plant diseases. The PKS gene cluster of an antimicrobial compound, phomenoic acid, has recently been identified in the canola pathogen *Leptosphaeria maculans* (Elliott et al. 2013). Elliott et al. (2013) identified that *P. nodorum* encodes a close PKS homologue of *L. maculans pks2*, which is SNOG_04868 (90% identity to PKS2). Along with the presence of phomenoic acid tailoring genes homologues in the SNOG_04868 cluster, this suggests a high likelihood that *P. nodorum* also produces phomenoic acid (Supplemental Table 2, cluster 10). Interestingly, no phomenoic acid can be detected in *P. nodorum* under the same culture condition for *L. maculans*, showing that the regulation of this PKS gene differs from that in *P. nodorum* (Elliott et al. 2013).

SNOG_06676 shares 66% protein identity with the *Alternaria brassicicola* DEP5 (AbPKS5) HR-PKS, which synthesizes the histone deacetylase inhibitor depudecin (Wight et al. 2009). Depudecin appears to play a small role in the virulence of *A. brassicicola* on cabbage. Another HR-PKS SNOG_11272 shares 50% identity with *Botrytis cinerea* BcBOA9 involved in the production of the phytotoxin botcinic acid (Dalmais et al. 2011). It is unknown if *P. nodorum* SNOG_06676 and SNOG_11272 produce related phytotoxic molecules. Other similar HR-PKSs in *P. nodorum* may also be responsible for production of macrolide compounds such as the phytotoxic stagonolides from *Stagonospora cirsii* (Evidente, Cimmino, Berestetskiy, Andolfi, et al. 2008; Evidente, Cimmino, Berestetskiy, Mitina, 2008) (Supplemental Table 1).

The hybrid PKS-NRPSs are HR-PKSs that have a single NRPS module appended at the C-terminal. *P. nodorum* has one such hybrid PKS-NRPS, SNOG_00308. The closest homologue of SNOG_00308 is the cytochalasin (Figure 3) PKS CcsA (46% protein identity) from *Aspergillus clavatus* (Qiao, Chooi, et al. 2011). Qiao, Chooi, et al. (2011) identified homologues of *ccsC, ccsD, ccsE, ccsF* and *ccsG* in the *P. nodorum* genome clustering with SNOG_00308, suggesting that *P. nodorum* may produce related cytochalasin compounds (Supplemental Table 2, cluster 2). Cytochalasins are actin polymerization inhibitors and are known to be produced by plant-associated fungi, including dothidiomycete pathogens from the genera *Phoma, Aschochyta* and *Drechslera* (Scherlach et al. 2010) and from many other kinds of ascomycete fungi. Some cytochalasins have been reported to be phytotoxic (Evidente et al. 2002; Berestetskiy et al. 2008), while others have demonstrated that cytochalasins inhibit the actin-related plant defence mechanism, allowing non-host pathogens to penetrate plant cells (Kobayashi, Kobayashi, et al. 1997; Kobayashi, Yamada, 1997). However, involvement of cytochalasins in pathogenicity of *P. nodorum* has not been demonstrated. Compounds isolated from other *Stagonospora* species that may be produced by a PKS-NRPS include the tetramic acid antifungal, pramanicin (Schwartz et al. 1994) (Supplemental Table 1).

Mellein has been reported to be a major metabolite of *P. nodorum* along with the *O-*methyl and hydroxylated analogues (Devys et al. 1980). Mellein isolated from *Diplodia pinea* (=*Sphaeropsis sapinea*) was shown to be phytotoxic against pine and tomato (Cabras et al. 2006). Interestingly, mellein is also a metabolite of actinomycete bacteria, including *Saccharopolyspora erythraea*. The molecule has been shown to be produced by a PR-PKS in *S. erythraea* (Sun et al. 2012). So far, all characterized fungal PR-PKSs were shown to produce 6-methylsalicylic acid (Figure 3). Since no 6-methylsalicylic acid has been isolated from *P. nodorum* and SNOG_00477 is the only PR-PKS in *P. nodorum*, this makes SNOG_00477 a promising candidate PKS gene for mellein production (Yang et al. 2013). SNOG_14927 is another possible candidate PKS suggested by Yang et al. (2013); it has the identical domain architecture as SNOG_00477 and 6-methylsalicylic acid PR-PKSs. However, SNOG_14927 shares higher sequence similarity to other HR-PKSs, such as *F. verticillioides* Fum1p (Table 2), suggesting SNOG_14927 may have diverged from an ancestral HR-PKS more recently via losing its ER domain. It is to be determined which of the two PKS above is responsible for production of mellein and its derivatives in *P. nodorum*.

Besides the iterative type I PKSs typical to fungi, *P. nodorum* harbours a type III PKS gene (SNOG_09622), which shares 36% protein identity to *Neurospora crassa* 2ʹ-oxoalkylresorcylic acid synthase (ORAS) (Funa et al. 2007). Yang et al. suggested that SNOG_09622 along with the neighbouring HR-PKS gene SNOG_09623 may be responsible for the production (+)-4ʹ-methoxy-(2S)-methylbutyrophenone (Yang et al. 2013). Presumably, SNOG_09623 synthesizes the methylated reduced diketide as a starter unit, which is then incorporated by SNOG_09622 into (+)-4ʹ-methoxy-(2S)-methylbutyrophenone.

### Nonribosomal peptide synthetases (NRPSs)

A minimum NRPS module typically consists of an adenylation (A) domain, thiolation domain (T) and a condensation (C) domain. Based on the typical collinearity rule of NRPSs (Finking & Marahiel 2004), the number of modules in an NRPS usually hints at the number of peptide residues in its final product. The domain architecture of *P. nodorum* NRPSs and their corresponding homologues (closest characterized homologue and/or closest BLASTP homologue) are shown in Table 3. *P. nodorum* has a total of six NRPSs with more than two modules. Except for SNOG_14834, all of these multimodular NRPSs end with a C-terminal condensation domain. The C-terminal condensation domain (C_T_ domain) of fungal NRPSs has been shown to be involved in peptide cyclization (Gao, Haynes, et al. 2012); thus, these multimodular NRPSs in *P. nodorum* may be responsible for the production of cyclic peptides. We will discuss the possible function of the *P. nodorum* NRPSs below, beginning with the multimodular NRPSs, followed by the dimodular and monomodular NRPSs.

**Table 3.** Domain architecture of selected NRPSs encoded in *Parastagonospora nodorum* genome.

NRPS locus	Domain architecture	Closest BLAST hit(s)
Multimodular NRPSs*		
SNOG_01105	A-T-C-A-T-C-A-T-C-A-T-C-A-T-C	*Pyrenophora teres* PTT_10812 (79%)
SNOG_02134	A-T-C-A-T-C-A-T-C-T-C-T-C	*Pyrenophora teres* PTT_13461 (55%)
SNOG_09081	A-T-C-T-C-A-T-C-A-T-C-A-T-C-C	*Metarhizium anisopliae* MAA_01639 (41%)
SNOG_09488	C-A-T-C-A-T-C-A-T-C	*Sphaerulina musiva* SEPMU_134958 (35%)
SNOG_14098	A-T-C-A-T-C-A-T-C-A-T-C-A-C	*Metarhizium acridum* MAC_04974 (43%)
SNOG_14834	T-C-A-T-C-A-T-C-A-T-C-A-T-C-A-T	*Pyrenophora teres* PTT_10907 (66%)
**Dimodular NRPSs**		
SNOG_14923	A-T-C-A-T-C	*Bipolaris maydis* NPS5 (75%)
**Monomodular NRPSs**		
SNOG_03620	A-T-TE	*L. maculans* LEMA_P067200.1 (73%)
SNOG_03771	A-T-R	*Pyrenophora teres* PTT_06870 (70%)
SNOG_04863	C-A	*Pyrenophora teres* PTT_16992 (62%)
SNOG_07021	A-T-C	*Pyrenophora teres* PTT_13470 (69%)
SNOG_07126	A-T-R-R	*L. maculans* Maa1 (85%)
SNOG_14368	A-T-C-T-T-C	*L. maculans* LEMA_P070680 (71%)

Notes: NRPS domain abbreviation – A, adenylation; T, thiolation; C, condensation; TE, thioesterase, R, reductase. See Finking and Marahiel (2004) review for detail description of the NRPS functional domains. *For multimodular NRPSs, the percentage protein identity values shown reflect the portion of the protein that has the highest match to the homologue (i.e. the value taken from a BLAST search) and may not represent 100% head-to-tail coverage (see ‘Methods’).

**Figure 4.**
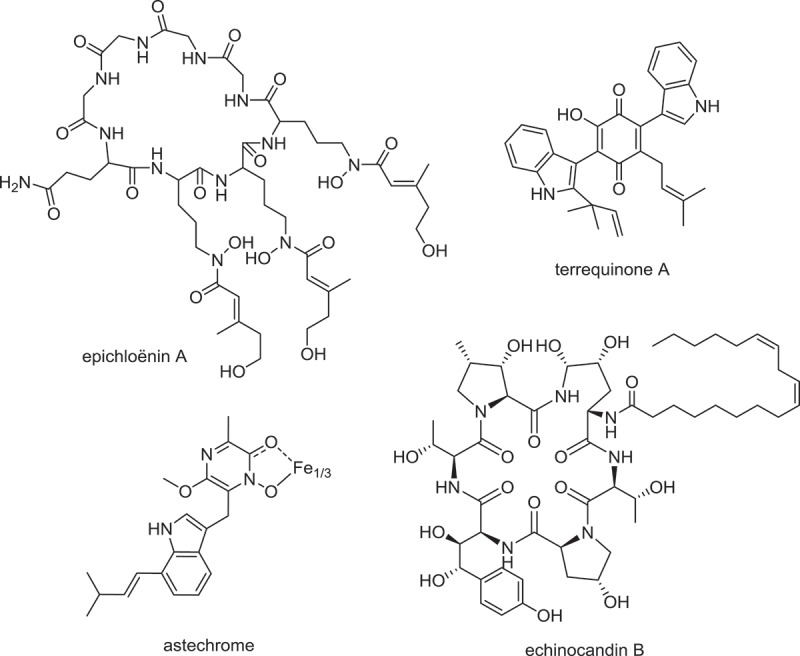
Nonribosomal peptide compounds synthesized by putative homologues of *Parastagonospora nodorum* NRPSs.

Siderophores are iron-sequestering cyclic peptides that play significant roles in virulence and fungal-host interactions (Johnson 2008). Interestingly, almost all known hydroxamate siderophore-synthesizing NRPSs do not follow the collinearity rule typical of NRPSs, but the iterative use of A domains (activating an identical amino acid more than once in an NRPS catalytic cycle) is the norm, with additional T-C domains as partial modules that extend the NRP products beyond the number of complete A-T-C modules in the NRPSs (Schwecke et al. 2006; Johnson 2008). SNOG_02134 is one such NRPS in *P. nodorum*, which consists of three A-T-C modules and two additional T-C partial modules. Recently, the SidN homologue from *Epichloe festucae* has been shown to be responsible for the production of a new siderophore, epichloënin A (Figure 4) (Johnson et al. 2013). To examine if *P. nodorum* encodes NRPSs with A domain that activate N^δ^-acyl-N^δ^-hydroxy-L-ornithine, the hydroxamate-containing residues commonly found in siderophores, we performed a BLAST search of the *P. nodorum* genome with the SidN-A3 domain as the query sequence (Lee et al. 2010). The closest blast hits to SidN-A3 domain is the first and third domain of SNOG_02134 NRPS, which shared 32% and 33% identity respectively with SidN-A3 domain. A putative L-ornithine N5-oxygenase known to be involved in hydroxamate siderophore biosynthesis (Hissen et al. 2005) is also encoded in the SNOG_02134 gene cluster (Supplemental Table 2, cluster 5). Thus, it is worth investigating whether SNOG_02134 encodes for hydroxamate siderophore biosynthesis in *P. nodorum*.

SNOG_14834 encodes a four module NRPSs with a T domain at the N-terminus. Fungal NRPSs with an N-terminal T domain have been shown to be involved in the biosynthesis of bioactive lipopeptides, such as emericellamide (Chiang et al. 2008), echinocandin (Figure 4) (Cacho et al. 2012) and pneumocandin (Chen et al. 2013). Interestingly, *Pyrenophora teres* f. *teres* genome contains an orthologue of SNOG_14834 (PTT_10907, 66% protein identity), however it appears that the orientation of the last two domains of the NRPSs are in reverse order compared to SNOG_14834, with PTT_10907 terminates with a C domain. It is yet to be determined if SNOG_14834 and PTT_10907 encode for cyclic lipopeptides. One of the *P. nodorum* multimodular NRPS genes (SNOG_09488) is located in close vicinity of a HR-PKS gene (SNOG_09490), suggesting the two genes may be part of a SM gene cluster (Supplemental Table 2, cluster 21). SNOG_09488 NRPS begins with an N-terminal C domain. Although such NRPSs have not been characterized in fungi, bacterial NRPSs with N-terminal C domain (or starter C domain) have been shown to be involved in biosynthesis of lipopeptides, where the starter C domain is responsible for incorporation of fatty acid or polyketide derived acyl-chain as a NRPS starter unit (Chooi & Tang 2010; Kraas et al. 2010, 2012). Therefore, it may be possible that the SNOG_09488 NRPS utilizes the polyketide chain synthesized by SNOG_09490 HR-PKS as starter unit to produce similar lipopeptide compound(s).

Septorine and its N-methoxy derivatives are phytotoxic pyrazine-containing molecules that have been isolated from *P. nodorum* (Devys et al. 1978, 1982, 1992). Thiopyrazine-synthesizing monomodular NRPS from *Aspergillus terreus* has been characterized previously (Qiao, Zhou, et al. 2011). However, the two oxygen substitutions in addition to the acyl substitutions at the pyrazine ring of septorines suggest that they are more similar to another class of NRPS molecule called diketopiperazine, which include sirodesmin (Gardiner et al. 2004), gliotoxin (Balibar & Walsh 2006; Cramer et al. 2006), and brevianamide (Maiya et al. 2006). More recently, another group of tryptophan-derived diketopiperazines, including hexadehydro-astechrome, with similar methoxy substitution as septorine has been isolated from *Aspergillus fumigatus* (Yin et al. 2013). Hexadehydro-astechrome (Figure 4) has the tendency to form iron complex and could be acting as a siderophore in *A. fumigatus*. As septorine, like most diketopiperazines natural products, is derived from the condensation of two amino acids (a tyrosine and an l-isoleucine), it is most likely synthesized by a dimodular NRPS. SNOG_14923 appears to be the only dimodular NRPS in *P. nodorum*.

Many single modular NRPS-like proteins do not contain the typical A-T-C module architecture, but often consist of A-T didomain followed by a variety of C-terminal domains (Bushley & Turgeon 2010). These monomodular NRPS-like proteins may or may not be involved in SM biosynthesis; for example, the aminoadipate reductase LYS2 in lysine biosynthesis pathway (terminates with thioester reductase domain). One of the monomodular NRPS that may be involved in SM biosynthesis is SNOG_03620 (33% identity to *A. nidulans* TdiA), which terminates with a thioesterase (TE) domain. These A-T-TE monomodular NRPSs are known to synthesize 1,4-benzoquinone compounds from condensation of two identical α-keto acids derived from amino acids; they include SM such as terrequinone (Figure 4) by TdiA (Balibar et al. 2007; Schneider et al. 2007) and atromentin by AtrA (Schneider et al. 2008; Wackler et al. 2012). More recently, such A-T-TE NRPS-like enzyme AN3396 (33% identity to SNOG_03620) has also been shown to produce a furanone compound, named microperfuranone (Yeh et al. 2012). No such α-keto acid-derived benzoquinone of furanone compounds have been discovered in *P. nodorum* so far. Interestingly, the canola pathogen *L. maculans* harbours a close homologue of SNOG_03620 (73% protein identity) with identical domain architecture. Besides SNOG_03620, most *P. nodorum* monomodular NRPSs appear to be conserved among the plant pathogens of the dothideomycete class as well (Table 3). Conservation of monomodular NRPSs with unknown functions among the dothideomycete was previously noted and it was proposed that some of these may play important roles in cellular metabolism (Bushley & Turgeon 2010).

### Terpene synthases (TSs) and prenyltransferases (PTases)

Some terpenoid compounds are known to play significant roles in plant pathogenic fungi. The isoprenoid backbones of these compounds are synthesized by terpene synthases (TSs). The classification of various terpene synthases and their catalytic mechanisms have recently been reviewed (Gao, Honzatko, et al. 2012). Notable plant pathogen examples where the TSs in the pathway have been characterized include trichothecene family of mycotoxins (Cane et al. 1995; Rynkiewicz et al. 2002), and the non-host-specific phytotoxin botrydial (Pinedo et al. 2008). Although no terpenoid SMs have been reported from *P. nodorum*, the genome revealed that it encodes three putative sesquiterpene synthases (class I), which include SNOG_03562, SNOG_04807, and SNOG_10024. The diterpene plant hormone gibberellins are known to be produced by a *Phaeosphaeria* sp. strain L487 (Supplemental Table 1), where the *ent*-kaurene synthase that catalyses the key step in gibberellin biosynthesis has been characterized (Kawaide et al. 1997). A BLAST search using the *ent-*kaurene synthase from *Phaeophaeria* sp. L487 and *Gibberella fujikuroi* (Tudzynski et al. 1998) did not detect any significant homologue in *P. nodorum* suggesting that this fungus is incapable of producing gibberellins. Nevertheless, a gene (SNOG_120607) encoding a class II diterpene cyclase can be found in the *P. nodorum* genome adjacent to a cytochrome P450 monooxygenase (Supplemental Table 2, cluster 28). Thus, the gene cluster may be responsible for the production of a diterpene molecule.

Fungal aromatic prenyltransferases (PTases) belongs to the dimethylallyltryptophan synthase (DMATS) family are the key enzymes to the biosynthesis of many indole alkaloids (Li 2009). The most well-known examples among the plant-associated fungi are the ergot alkaloids (Wallwey & Li 2011). More recently, these DMATS-type PTases have also been implicated in biosynthesis of meroterpenoid compounds (polyketide-terpenoid hybrid compounds), such as viridicatumtoxin (Chooi et al. 2012) and prenyl xanthones (Sanchez et al. 2011). Two PTases were found in the *P. nodorum* genome, SNOG_06682, which clustered together with a NR-PKS gene (SNOG_06680), and SNOG_008527. Besides mycophenolic acid (Devys et al. 1980), which was mentioned above, no meroterpenoid or indole alkaloid compounds have been identified from *P. nodorum*. Two prenylated compounds that have been isolated from related species are spartinoxide and 4-hydroxy-3-prenyl-benzoic acid (Elsebai et al. 2010) (Supplemental Table 1).

Other than the DMATS-type PTases, *P. nodorum* also harbours five genes encoding UbiA-like membrane-bound PTases, which is responsible for transfer of long prenyl chains, such as the *E. coli* UbiA and *S. cerevisiae* COQ2 (p-hydroxybenzoate:polyprenyl transferase) of the ubiquinone pathway. UbiA-like PTases are also known to be involved in biosynthesis of SMs, such as the pyripyropene A (Itoh et al. 2010) and 3,5-dimethylorsellinic acid (DMOA)-derived meroterpenoids (Itoh et al. 2012; Lo et al. 2012). More recently, a novel membrane-bound class I terpene cyclase, which shares structural and sequence homology to UbiA-like PTases, has been shown to cyclise farnesyl-diphosphate to *β*-trans-bergamotene in the fumagillin pathway (Lin et al. 2013). Two of the five *P. nodorum* UbiA-like PTases (SNOG_00816 and 05304) showed overall high protein identity (>80%) with other ascomycetes, and are likely to be involved in primary metabolism. The other three UbiA-like PTases (SNOG_00008, 07120 and 09915) share lower similarity to those found in other ascomycete genomes and may be involved in SM biosynthesis. The SNOG_09915 gene is clustered with a HR-PKS gene (SNOG_09932) and maybe responsible for biosynthesis of a meroterpenoid of mixed polyketide-isoprenoid origin (Supplemental Table 2, cluster 23). On the other hand, the SNOG_07120 locus is in vicinity of a NRPS gene (SNOG_07126) and thus may be a terpene alkaloid gene cluster (Supplemental Table 2, cluster 15).

## Future perspectives

In summary, the whole-genome survey of the SM gene inventory of *P. nodorum* shows that the wheat pathogen harbours significant unexplored SM biosynthetic potential. However, none of the SM products from these gene clusters are known and only a handful of metabolites (representing only about one-tenth of the total number of gene clusters in *P. nodorum*) have been identified from this important wheat pathogen. Several SM backbone genes/gene clusters appear to be conserved across multiple plant pathogens; these SM genes may play important roles that are common to their pathogenic life style. Apart from uncovering novel SM virulence factors, the *P. nodorum* genome presents a unique opportunity for natural product discovery with some SM gene clusters potentially encoding analogues of known bioactive molecules amongst others that are novel.

## Methods

### *In silico* analysis of *P. nodorum* SM genes

*P. nodorum* PKS genes were retrieved from the *P. nodorum* genome on the NCBI GenBank and DOE-JGI database by using an arbitrary fungal KS domain (e.g. *P. aethiopicum* VrtA) as a BLASTP query. The PKS domains are predicted using the NCBI Conserved Domain Search and antiSMASH 2.0 (Blin et al. 2013). The SAT and PT domains of NR-PKSs not detected by the automated analyses above were determined by protein sequence alignment with *Aspergillus parasiticus* norsoloronic acid synthase (PksA) and manually search for the conserved motifs described in the previous work (Crawford et al. 2006, 2008). The closest characterized BLAST hits were retrieved by BLASTP program on NCBI website using *P. nodorum* PKSs as query sequences.

*P. nodorum* NRPS genes were retrieved from the *P. nodorum* genome by using an arbitrary fungal A domain (e.g. *P. aethiopicum* TqaA-A1domain) as a BLASTP query. The NRPS domains were predicted using the NCBI Conserved Domain Search and antiSMASH2.0 (Blin et al. 2013). Due to the large size of these proteins and their tendency to rearrange/truncate during evolution, a good head-to-tail pairwise alignment was not possible unless there was a close homologue with identical domain architecture. Thus, for quick and simple comparison, the percentage protein identity values was taken directly from the BLASTP analysis and they reflect the portion of the protein that has the highest match to the homologue and may not represent 100% head-to-tail coverage, especially those that shown a lower percentage identity scores. Nevertheless, the values are still useful for comparison as the portion of protein that has a good match is often the conserved domain (C, A and T domains) and may reflect the substrate specificities.

*P. nodorum* PTase and TS genes were retrieved from the genome by using an arbitrary fungal PTase (e.g. *P. aethiopicum* VrtC) and TS (e.g. trichodiene synthase and aristolochene synthase) respectively as BLASTP query.

A list of all the SM backbone genes retrieved using the methods above was generated and compared with the list of *P. nodorum* gene clusters predicted by SMURF (Khaldi et al. 2010). The loci where the SM backbone genes were not detected by SMURF were manually inspected for neighbouring biosynthetic genes on the DOE-JGI genome browser. These additional *P. nodorum* SM gene clusters were added to the list in Supplemental Table 2.
